# Anticancer Property of Digera muricata Leaf Extract: An In Vitro Study

**DOI:** 10.7759/cureus.49276

**Published:** 2023-11-23

**Authors:** Podhigai Selvi Varadarajan, Anitha Roy, D Jagadeswaran

**Affiliations:** 1 Neuroelectrophysiology, Saveetha College of Allied Health Sciences, Saveetha Institute of Medical and Technical Sciences, Saveetha University, Chennai, IND; 2 Pharmacology, Saveetha Dental College and Hospitals, Saveetha Institute of Medical and Technical Sciences, Saveetha University, Chennai, IND; 3 Renal Science and Dialysis Technology, Saveetha College of Allied Health Sciences, Saveetha Institute of Medical and Technical Sciences, Saveetha University, Chennai, IND

**Keywords:** mtt assay, ethanolic extract, mg-63, osteosarcoma cell line, digera muricata

## Abstract

Aim

The aim was to evaluate the anticancer potential of *Digera muricata *ethanolicleaf extract on MG-63 osteosarcoma cell lines.

Materials and methods

The anti-cancer properties of *Digera muricata *ethanolic leaf extract were evaluated on osteosarcoma cell lines using 3- (4,5-dimethylthiazol-2-yl)-2,5-diphenyltetrazolium bromide (MTT) assay, and the morphological changes in MG-63 cells were assessed after 24 hours using microscopic observation. Additionally, fluorescence microscopy was employed to evaluate the apoptotic changes after acridine orange/ethidium bromide (AO/EtBr) dual staining.

Results

The MTT assay revealed a dose-dependent cell death. The cell viability decreased with increase in concentrations of the extract, The cell viability was 89.98 ± 4.89 percentage at 25 μg/ml and 15.64 ± 3.64 percentage at 200 μg/ml concentrations. A concentartion of 116.95 μg/ml showed 50% inhibition (IC_50_). The morphological and dual staining studies also showed the extract's effectiveness in inducing apoptosis.

Conclusion

The ethanolic leaf extract of *D. muricata* could impart good antiproliferative activity in MG-63 cell lines. The extract could also induce apoptosis and hence, it may be considered as a potential anticancer agent for the development of drug formulation for the treatment of osteosarcoma.

## Introduction

Cancer is a common health problem and a leading cause of death worldwide [1]. Osteosarcoma is a potentially fatal and complex disease often seen in adolescent people [2]. Osteosarcoma is a common malignant bone tumour where tumour cells form immature bone and osteoid tissue, mostly in the long bones of the arms and legs [3-5]. The incidence is higher in males than females, and it can occur with or without underlying pathology [6]. Swelling, joint dysfunction, and local pain are common with it [2]. However, there have been significant improvements in treatment options and survivability due to research and trials. There are different therapeutic approaches for cancer diagnosis and treatment [7-9]. The common approaches include chemotherapy, radiotherapy, immunotherapy, enzyme therapy, antibiotics, and surgery [2]. However, the commonly used agents are cytotoxic with varying degrees of nausea, vomiting, and other complications, which are at times worse than the disease itself. Hence, much research is going on to tackle the various cancers with phytochemicals in order to minimise side effects and have more patient compliance [10]. The plant selected in the study is rich in phytochemicals responsible for various pharmacological activities. Moreover, the use of nutraceuticals in cancer chemotherapy has gained popularity due to the complexity of the disease and its global impact [11,12]. The main problem with osteosarcoma is the rapidity in tumour growth, metastasis to lungs, and delay in diagnosis leading to a high mortality rate. The current treatment modality for osteosarcoma is adjuvant chemotherapy and surgery [13].

*Digera muricata* (*D. muricata*) family *Amaranthaceae* is a herb that grows up to 20-70 cm in the Western Himalayas, mostly in moist areas with an altitude of 1000 m [14]. It has many therapeutic uses. It has antimicrobial, anthelmintic, antioxidant, antidiabetic, hepato-protective, and nephron-protective properties [15]. Its roots are used as a galactagogue, and the leaves are used for treating kidney stones [16-20]. The flower, seeds, and extract of the plant are used to treat urinary discharges [17,20].

*D. muricata* is rich in phytochemicals such as flavonoids, alkaloids, terpenoids, tannins, saponins, anthraquinone, coumarins, cardiac glycosides, α-spinasterol, β-spinasterol, rutins, hyperoside flavonoids. It is a rich source of vitamins such as thiamine, ascorbic acid, and β-carotene [21]. Being a plant with rich phytochemicals, the anticancer property of *D. muricata* ethanolic leaf extract was explored in the present study.

## Materials and methods

Reagents

The reagents used for the study [3-(4,5-dimethythiazol-2-yl) 2,5-diphenyl tetrazolium bromide (MTT), 4′,6-diamidino-2-phenylindole (DAPI), acridine orange/ethidium bromide (AO/EtBr) were purchased from Sigma Chemical Pvt Ltd, USA. Dulbecco's Modified Eagle Medium (DMEM), Phosphate Buffered Saline (PBS), Trypsin-ethylenediaminetetraacetic acid (EDTA), and fetal bovine serum (FBS), were purchased from Gibco, Canada. Acridine orange (AO), ethidium bromide (EtBr), and dimethyl sulfoxide (DMSO). All other chemicals used were extra pure of molecular grade and were purchased from SRL, India.

Cell line maintenance

This study has used an osteosarcoma cell line (MG-63) purchased from the National Centre for Cell Science (NCCS), Pune, India. The cells were grown in T25 culture flasks containing DMEM supplemented with 10% FBS and 1% antibiotics. Cells were maintained at 37◦C in a humidified atmosphere containing 5% CO2. Upon reaching confluency, the cells were trypsinized and passaged [22].

Preparation of *Digera muricata* ethanolic leaf extract

*D. muricata leaf powder *obtained from Imcops Hospital in Chennai, India, was used for the present study. To obtain the ethanolic extract, the leaf powder was mixed with 95% ethanol, filtered, and stored at 4°C. Fifty grams of *D. muricata* leaf powder was soaked in 500 mL of 95% ethanol and kept at room temperature for thre days in a static condition. Then the solution was filtered with crude filter paper followed by Whatman paper. The fine filtrate was subjected to rotary evaporation, and after that, 3 g of the material was obtained. The total ethanol extract was concentrated in a vacuum evaporator and immediately stored at 4˚C.

Cell viability (MTT) assay

The cell viability of *D.muricata *ethanolic leaf extract-treated MG-63 cells was assessed by MTT assay [22]. The assay is based on the reduction of soluble yellow tetrazolium salt to insoluble purple formazan crystals by metabolically active cells. MG-63 cells were plated in 96 well plates at a concentration of 5x10³ cells/well 24 hours after plating, cells were washed twice with 100 μl of serum-free medium and starved by incubating the cells in serum-free medium for three hours at 37ºC. After starvation, cells were treated with different concentrations *D. muricata *ethanolic leaf extract for 24 hours. The control cells were treated with 0.1% ethanol. At the end of treatment, the medium from control and *D. muricata*leaf extract treated cells were discarded and 100 μl of MTT containing DMEM (0.5 mg/ml) was added to each well. The cells were then incubated for 4h at 37ºC in the CO_2_ incubator.

The MTT-containing medium was then discarded and the cells were washed once with PBS. Then the formazan crystals formed were dissolved in dimethyl sulfoxide (100 μl) and incubated in the dark for an hour. Then the intensity of the color developed was assayed using a Micro enzyme-linked immunosorbent assay (ELISA) plate reader at 570 nm. The number of viable cells was expressed as a percentage of control cells cultured in a serum-free medium. Cell viability in the control medium without any treatment was represented as 100%. The cell viability is calculated using the formula: % cell viability = [A570 nm of treated cells/A570 nm of control cells] ×100.

Acridine orange (AO)/ethidium bromide (EtBr) dual staining

The mode of cell death was determined by acridine orange (AO)/ethidium bromide (EtBr) dual staining. Fluorescence microscopy was used to evaluate the apoptotic changes in cells treated with *D. muricata* ethanolic leaf extract after dual staining. The effect of *D. muricata *ethanolic leaf extract in MG-63 cell death was also determined by AO/EtBr dual staining as described previously [23]. The cells were treated with *D. muricata* ethanolic leaf extract for 24 hours and then the cells were harvested and washed with ice-cold PBS. The pellets were resuspended in 5 µl of acridine orange (1 mg/mL) and 5 µl of EtBr (1 mg/mL).

Statistical analysis

One-way ANOVA followed by Student's-t-test was carried out by SPSS software, Version 9.05 (SPSS Inc., Chicago, IL, USA). The data was collected in triplicate and its mean ± SD was tabulated with a significance of p < 0.05.

## Results

**Figure 1:**
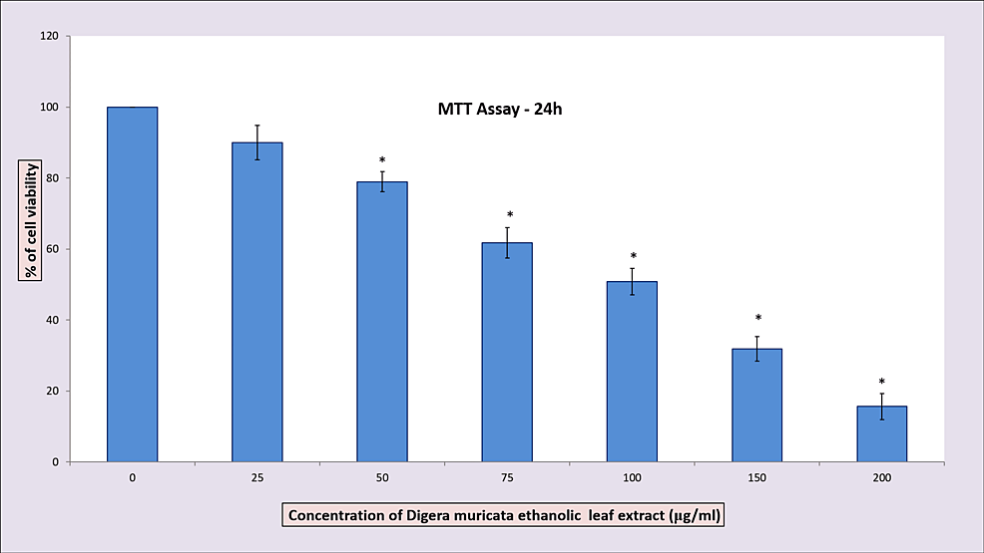
MTT assay The cytotoxic effects of *D. muricata* ethanolic leaf extract on osteosarcoma cells. Cells were treated with different concentrations (25, 50, 75, 100, 150, and 200 μg/ml) for 24 hours, and cell viability was evaluated by MTT assay. Data are shown as means ± SD (n = 3). * compared with the control blank group, p < 0.05. MTT: 3-(4,5-dimethythiazol-2-yl) 2,5-diphenyl tetrazolium bromide

The results of the study indicate that *D. muricata* ethanolic leaf extract exhibited cytotoxic activity against MG-63 osteosarcoma cell lines, and the viability was reduced with an increase in the dose of the extract. The percentage inhibition was 89.98 ±4.89, 78.98 ± 2.79, 61.77 ± 4.24; 50.86 ± 3.73, 31.86 ±3.49 15.64 ± 3.64 at 25 at 25, 50, 75, 100, 150 and 200 µg/ml. Figure 1 illustrates the dose-dependent decrease in cell viability, with an IC_50_ value of 116.95 μg/ml. The IC_50_ value was calculated using the formula Y = MX-CE.

**Figure 2:**
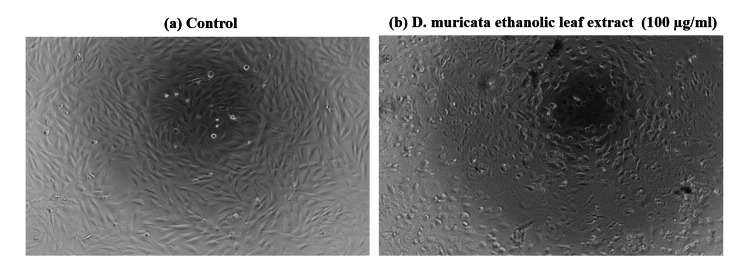
Morphology of osteosarcoma cells Inverted phase contrast microscopic images a. Control osteosarcoma cells without extract treated, and b. Osteosarcoma cells treated with *D. muricata *ethanolic leaf extract (100 μg/ml concentration) for 24 hrs.

**Figure 3:**
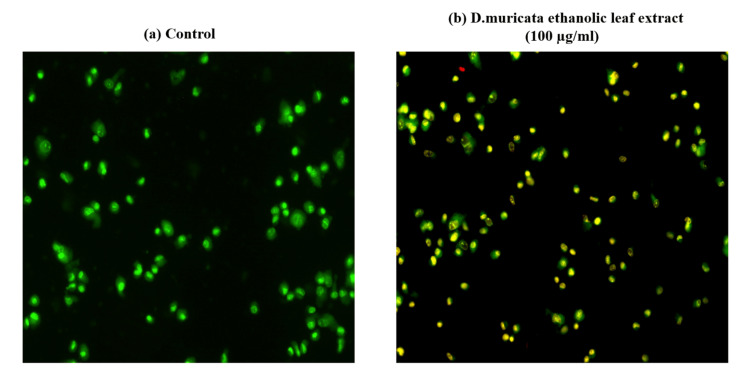
Fluorescence microscopic images of osteosarcoma cells a. Control osteosarcoma cells without extract treatment and b. Osteosarcoma cells treated with *D. muricata* ethanolic leaf extract (100 µg/ml) for 24 hours.

As the treated cells showed 50% inhibition around 100 μg/ml, this particular concentration was used for the morphological study as well as for fluorescence image. The impact of the treatment with *D. muricata* ethanolic leaf extract at a concentration of 100 μg/ml on the appearance of the osteosarcoma cell lines is shown in Figure 2. The images obtained by fluorescence microscopy following AO/EtBr dual staining of the MG-63 osteosarcoma cell line with and without test extract for 24 hrs are presented in Figure 3, indicating condensed chromatin and nuclear fragmentation in the treated cells.

## Discussion

Cell line studies can help to assess the anticancer potential without compromising animals and not being invasive. Moreover, it can open a gateway to identify the candidate with anticancer potential and further explore the drug for specific cancer. Different cell lines are used to explore the anticancer activity of different phytochemicals.

*D. muricata* ethanolic leaf extract’s effect in the viability of MG-63 cell line using MTT assay detects formazan product intensity resulting from MTT reduction by succinate dehydrogenase in the mitochondria [20]. The MTT assay involves the metabolic conversion of tetrazolium salt to coloured formazan crystals in living cells [22,23]. In this study, the viability of MG-63 cells treated with *D. muricata* ethanolic leaf extract at concentrations ranging from 25 μg/ml to 200 μg/ml. The results from the MTT assay showed that the cell viability decreased with an increase in extract concentration. The incubation of MG-63 cells with extract caused damage to the cells in a dose-dependent manner. The results showed a significant cytotoxic effect and the IC_50_ dose was 116.95 μg/ml.

The appearance of the osteosarcoma cell lines treated with extract at the same concentration exhibited observable changes compared to the control cells under an inverted phase contrast microscope. The AO/EtBr dual staining technique is an effective method to determine the viability of cells. Acridine orange can enter both living and non-living cells and produce green and red fluorescence on DNA and RNA, respectively. Ethidium bromide is taken up by non-living cells without membrane integrity, which have DNA and show red fluorescence. Similarly, early and late apoptotic cells exhibit green nuclei and orange to red nuclei with chromatin fragments, respectively. Necrotic cell nuclei do not show condensed chromatin [23].

In this study, the AO/EtBr dual staining of the MG-63 osteosarcoma cell line exposed to *D. muricata* leaf extract for a period of 24 hours showed distinct differences, inferring the effectiveness of the extract. The nuclei of the treated cells stained with AO/EtBr showed condensed chromatin and nuclear fragmentation.

Previous studies by Usmani et al. (2014) have reported the cytotoxic effect of aqueous and methanolic extract of D. muricata when used in a range of 25 μg/ml to 250 μg/ml in both human cell lines of the cervix and lungs. The flavonoids present in the methanolic extract may be responsible for the antiproliferative effect [24]. *D. muricata *ethanolic leaf extract could produce cytotoxicity on MG-63 osteosarcoma cells by inducing apoptosis. The presence of flavonoids in the extract and its fractions may be responsible for the observed anticancer activity. Other plant extracts, such as the methanolic extract of *Cissus quadrangularis* and *Sterculia foetida* seed, have also been evaluated for their anticancer activity on MG-63 osteosarcoma cells [11,24,25]. The previous literature has shown that 5-8 gm of *D. muricata* thrice daily is safe and effective for various diseases [26]. The present study also suggests the potential antiproliferative effect of *D. muricata* ethanolic leaf extract against the MG-63 osteosarcoma cell line.

Limitations

The exact phytochemical responsible for the anticancer activity from the ethanolic leaf extract of *D. muricata* was not isolated in order to find the exact molecular mechanisms behind the cytotoxic effect of *D. muricata*. Hence, the future plan is to isolate the exact phytochemical responsible for the anticancer activity and to further explore the apoptosis pathway and also to find any toxicity with the extract so that it can be safely used in humans.

## Conclusions

A dose-dependent cytotoxic effect of *D. muricata* ethanolic leaf extract was observed. Higher concentrations of the extract showed better anti-proliferative activity. The morphological and dual staining studies conducted on the cells have also provided evidence of the extract's effectiveness in inducing apoptosis. Therefore, the extract could be considered a potential anti-cancer agent for treating osteosarcoma. However, isolating the active principle and conducting further studies could help in understanding the exact molecular mechanisms behind the cytotoxic effect of *D. muricata*.
